# MeGLYI-13, a Glyoxalase I Gene in Cassava, Enhances the Tolerance of Yeast and *Arabidopsis* to Zinc and Copper Stresses

**DOI:** 10.3390/plants12193375

**Published:** 2023-09-25

**Authors:** Ruimei Li, Fenlian Tang, Yannian Che, Alisdair R. Fernie, Qin Zhou, Zhongping Ding, Yuan Yao, Jiao Liu, Yajie Wang, Xinwen Hu, Jianchun Guo

**Affiliations:** 1 Key Laboratory of Biology and Genetic Resources of Tropical Crops, Institute of Tropical Bioscience and Biotechnology, Chinese Academy of Tropical Agricultural Sciences, Haikou 571101, China; liruimei@itbb.org.cn (R.L.); 19095131210051@hainanu.edu.cn (F.T.); 22110710000011@hainanu.edu.cn (Y.C.); 21210710000025@hainanu.edu.cn (Q.Z.); 21220951310010@hainanu.edu.cn (Z.D.); yaoyuan@itbb.org.cn (Y.Y.); liujiao@itbb.org.cn (J.L.); wangyajie@itbb.org.cn (Y.W.); 2 Key Laboratory for Biology and Genetic Resources of Tropical Crops of Hainan Province, Hainan Institute for Tropical Agricultural Resources, Haikou 571101, China; 3 College of Tropical Crops, Hainan University, Haikou 570228, China; 4 Root Biology and Symbiosis, Max-Planck-Institute of Molecular Plant Physiology, Am Muhlenberg 1, 14476 Potsdam-Golm, Germany; fernie@mpimp-golm.mpg.de

**Keywords:** glyoxalase, cassava, heavy metal, zinc, copper, overexpression, yeast, *Arabidopsis*, subcellular localization

## Abstract

Although zinc and copper are the two essential nutrients necessary for plant growth, their excessive accumulation in soil not only causes environmental pollution but also seriously threatens human health and inhibits plant growth. The breeding of plants with novel zinc or copper toxicity tolerance capacities represents one strategy to address this problem. Glyoxalase I (GLYI) family genes have previously been suggested to be involved in the resistance to a wide range of abiotic stresses, including those invoked by heavy metals. Here, a *MeGLYI-13* gene cloned from a cassava SC8 cultivar was characterized with regard to its potential ability in resistance to zinc or copper stresses. Sequence alignment indicated that *MeGLYI-13* exhibits sequence differences between genotypes. Transient expression analysis revealed the nuclear localization of MeGLYI-13. A nuclear localization signal (NLS) was found in its C-terminal region. There are 12 Zn^2+^ binding sites and 14 Cu^2+^ binding sites predicted by the MIB tool, of which six binding sites were shared by Zn^2+^ and Cu^2+^. The overexpression of *MeGLYI-13* enhanced both the zinc and copper toxicity tolerances of transformed yeast cells and *Arabidopsis* seedlings. Taken together, our study shows the ability of the *MeGLYI-13* gene to resist zinc and copper toxicity, which provides genetic resources for the future breeding of plants resistant to zinc and copper and potentially other heavy metals.

## 1. Introduction

Given that heavy metal pollution has had, and will continue to have, a serious impact on the environment and plant, animal and microbial life, leading to the destruction of ecosystems, heavy metal pollution is attracting increasing worldwide attention [1]. There are many reasons for heavy metal pollution in farmland soil during agricultural production, including the use of wastewater for soil irrigation, the application of fertilizers, the spraying of pesticides, herbicides and so on [2]. Especially, the frequent and extensive use of various zinc (Zn)- and copper (Cu)-containing fertilizers and fungicides has resulted in the continuous accumulation of Zn and Cu in farmland soils [3]. Both Zn and Cu are essential micronutrients for plant growth and development. However, when their concentrations are too high, they result in toxicity to organisms [4,5]. 

Zinc is an essential nutrient for plants, because it is an important component of key protein structures and catalytic sites [6,7]. In plants, zinc has a significant effect on reactive oxygen metabolism and regulating defense reactions and signals [8,9]. Zinc deficiency reduces leaf biomass, increases ROS accumulation and affects plant hormone levels and TCA-related enzyme activities [9]. The leaves of Zn^2+^-deficient plants were notably yellow, and the levels of iron in their branches increased [6]. Zinc deficiency results in stunted growth, reduced tillers and low productivity of rice plants, with brown spots and stripes on the leaves [10]. Moreover, concerning zinc deficiency, some enzymes such as alkaline phosphatase (ALP), alcohol dehydrogenase (ADH) and polyphenol oxidase (PPO) activity decreased, while guaiacol peroxidase (GPX) activity increased [11]. Zinc toxicity also induces reactive oxygen species (ROS) accumulation [9,12]. Zinc toxicity additionally significantly reduces plant biomass, inhibits the accumulation of other metal ions, reduces the photosynthetic rate and renders plants more susceptible to disease [12].

Copper is an essential nutrient for the normal metabolism of aerobic life. As a cofactor of various redox enzymes, copper plays a key role in redox catalysis. Copper is also involved in the signal regulation, protein transport and metabolism of other metals in cells [13,14,15]. Copper deficiency in the growth environment additionally results in adverse effects on plant growth and development, such as inhibiting vegetative growth and reproductive development, causing leaf phenotypic distortion and color change, affecting pollen and embryo development, reducing crop yield and affecting cell wall formation and water transport [13]. Copper deficiency also affects photosynthesis and respiration in plants [16,17]. However, more copper is not always better, and more than the required levels can be toxic to the plant. Plant exposure to high concentrations of copper results in physiological disorder and dwarf phenotypes [18,19,20]. The excessive copper also inhibits root growth and alters root morphology [21]. The level of toxic ROS increases; the cell structure is damaged and the photochemical efficiency, chlorophyll content, stomatal conductance and transpiration rate are also affected in plants exposed to too-high levels of copper [18,19]. 

To cope with the damage of abiotic stress, plants have evolved a series of complex plant protection mechanisms to mitigate external damage. Some recent studies have found that the glyoxalase system is involved in plant protection against heavy metals by removing the excess methylglyoxal (MG) [22,23,24]. Glyoxalase I (GLYI) is the first enzyme in the MG detoxicant pathway [25]. The increased Cu tolerance in rice is accompanied by the activation of glyoxalase I and II and the decrease in the methylglyoxal content [24]. Similarly, it has been found that, in mung beans, glyoxalase was upregulated to reduce the Cu phytotoxicity [22]. The heterologous expression of the wheat *TaGLYI* gene in tobacco has improved the tolerance of transgenic tobacco to heavy metal Zn^2+^ stress [26]. Transgenic tobacco heterologously expressing the daylily *HfGLYI-1* is tolerant to abiotic stress at high concentrations of ZnCl_2_ [27]. Glyoxalase is also involved in the defense of plants against other heavy metal stress, such as nickel (Ni), chromium (Cr), iron (Fe), arsenic (As), vanadium and cadmium (Cd) [23,26,28,29,30,31,32,33,34,35]. 

Cassava (*Manihot esculenta*) is a woody shrub that accumulates considerable quantities of starch in its tuberous roots. It is not only a food crop to ensure world food security but also as a high-quality raw material for the production of clean bioenergy. A previous study indicated that cassava can be grown in heavy metal (Cd, Cu, Pb and Zn)-contaminated fields without affecting its biomass production [36]. This indicated that cassava has a strong resistance to heavy metal stress. Therefore, mining heavy metal resistance genes in cassava is of great value to improve heavy metal resistance in cassava and other plants. We have previously identified 19 putative *GLYI* genes from cassava, in which *MeGLYI-13* was found to play important roles in the resistance to Fe^3+^ stress [33]. However, whether the *MeGLYI-13* gene is involved in other heavy metal stresses, particularly Zn and Cu toxicity, remains unclear. Further study of *MeGLYI-13* sequences, structural characteristics, protein subcellular localization and tolerance to Zn and Cu stress is of great significance to broaden our understanding of the function of the *MeGLYI-13* gene and its potential application in modern agriculture.

## 2. Results

### 2.1. MeGLYI-13 Gene Sequence Analysis

The *MeGLYI-13* gene was cloned from the genome of the SC8 cassava cultivar using the Manes.12G054100 sequence retrieved from the AM560-2 cultivar as a reference (*Manihot esculenta* v6.1 dataset). Its cDNA length is 495 bp, and it encodes a protein of 164 amino acids in length. It has 99.3% similarity with the Manes.12G054100 sequence from cassava variety AM560-2 in the cassava genome database. There is one base difference between MeGLYI-13 in SC8 and Manes.12G054100 in AM560-2, which is “^205^C” in SC8 and “^205^T” in AM560-2. At the protein level, this corresponds to ”^69^P” in SC8 but “^69^S” in AM560-2 (Figure S1). The *MeGLYI-13* gene from SC8 was registered in NCBI GenBank (MW413417). 

### 2.2. Prediction of Zn^2+^ and Cu^2+^ Binding Sites in MeGLYI-13 Protein Sequence

**Figure 1:**
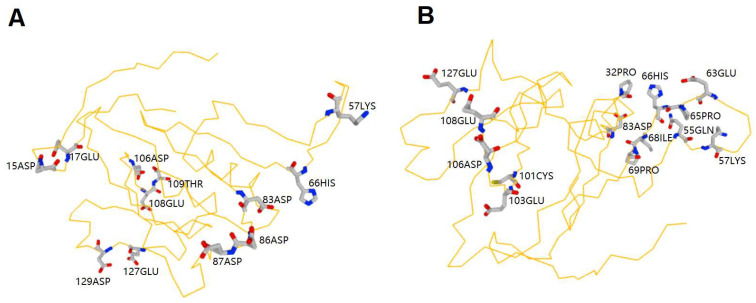
The Zn^2+^ and Cu^2+^ ion binding site analysis in the MeGLYI-13 amino acid sequence. (**A**) The Zn^2+^ binding sites in MeGLYI-13. (**B**) The Cu^2+^ binding sites in MeGLYI-13.

In order to understand the potential of MeGLYI-13 to bind zinc and copper ions, we predicted the Zn^2+^/Cu^2+^ ion binding sites in its protein sequence. The results showed that there were 12 Zn^2+^ binding sites and 14 Cu^2+^ binding sites in the MeGLYI-13 amino acid sequence. In addition, six sites: 57LYS, 66HIS, 83ASP, 106 ASP, 108GLU and 127GLU bound to both Zn^2+^ and Cu^2+^ (Figure 1).

### 2.3. MeGLYI-13 Protein Was Localized in the Nucleus

**Figure 2:**
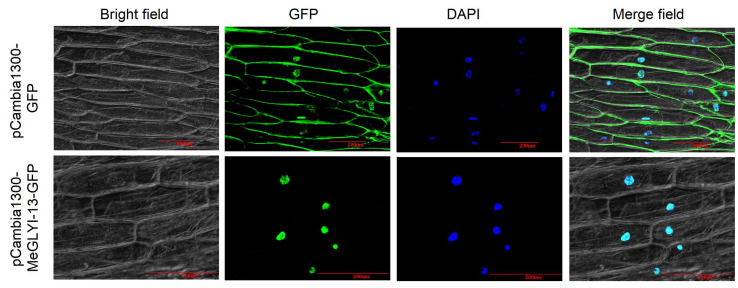
Subcellular localization of MeGLYI-13 in onion epidermal cells.

With the intention of assessing the localization of the MeGLYI-13 protein, pCambia1300-MeGLYI-13-GFP and pCambia1300-GFP plasmids were transformed into onion epidermal cells by *Agrobacterium tumefaciens* infection. The green fluorescent signal could be clearly observed in the cytoplasm and nucleus following infection with the control vector, whereas the fluorescent signal could only be detected in the nucleus after infection with the pCambia1300-MeGLYI-13-GFP vector. The nuclear localization was confirmed by DAPI staining (Figure 2). 

### 2.4. The Ability of Yeast to Tolerate Cu^2+^ and Zn^2+^ Toxicity Is Enhanced by MeGLYI-13

**Figure 3:**
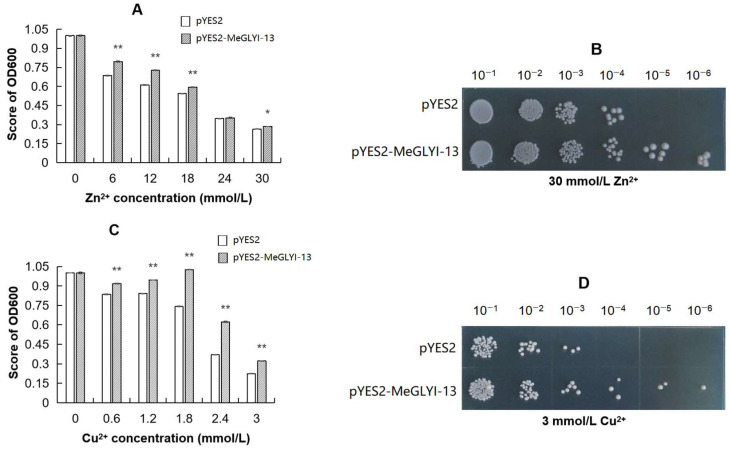
Overexpression of *MeGLYI-13* enhanced the yeast resistance to Zn^2+^ and Cu^2+^ toxicity. (**A**) Growth activity of yeast after different concentrations of Zn^2+^ treatments. (**B**) The growth of *MeGLYI-13* transgenic yeast and empty yeast cells after the 30 mmol/L Zn^2+^ treatment. (**C**) Growth activity of yeast after different concentrations of Cu^2+^ treatments. (**D**) The growth of *MeGLYI-13* transgenic yeast and empty yeast cells after the 3 mmol/L Cu^2+^ treatment. The error lines represent ±SD, *n* = 3; * signals a significant difference of *p* ≤ 0.05 and ** *p* ≤ 0.01.

The growth characteristics of INVSc1 yeast, containing the pYES2-*MeGLYI-13* plasmid or pYES2 control vector, respectively, treated by Zn^2+^ or Cu^2+^, were compared. The pYES2-*MeGLYI-13*-transformed yeast showed better growth ability than the control yeast after the Zn^2+^ and Cu^2+^ treatments (Figure 3). No colonies appeared after 10^−5^ dilution of the yeast transformed by the control vector after the Zn^2+^ treatment. However, under the same conditions, the pYES2-*MeGLYI-13*-transformed yeast could still grow after 10^−6^ dilution. After treatment with Cu^2+^, the growth of the 10^−3^ dilution control yeast was completely suppressed, whereas the growth of the 10^−6^ dilution pYES2-*MeGLYI-13* yeast was not completely suppressed (Figure 3). These results indicated that overexpression of the *MeGLYI-13* gene raised the resistance of INVSc1 yeast to both Zn^2+^ and Cu^2+^ toxicity.

### 2.5. Expression of MeGLYI-13 in Arabidopsis

**Figure 4:**
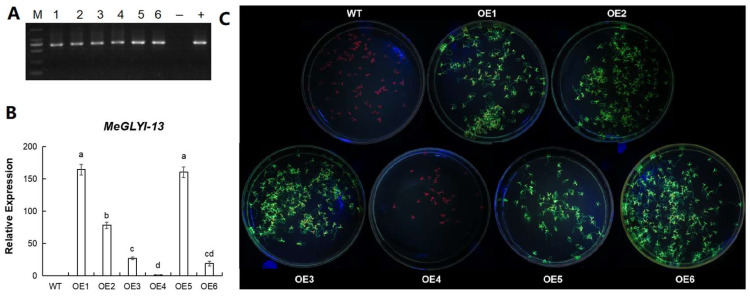
Generation of *MeGLYI-13* overexpressing *Arabidopsis*. (**A**) PCR analysis of transgenic *Arabidopsis*. M, DL2000 DNA Marker; 1~6, the transgenic *Arabidopsis* lines; +, the positive control (pCambia1300-MeGLYI-13-*GFP* vector plasmid); −, the negative control (wild type *Arabidopsis*). (**B**) Expression analysis of the different transgenic lines. WT, wild type Arabidopsis; OE1~OE6, the transgenic *Arabidopsis* lines. The error lines represent ±SD, *n* = 3; the character on the top of each bar represents a significant difference. (**C**) Fluorescence detection of the different transgenic lines. WT, wild type *Arabidopsis*; OE1~OE6, the transgenic *Arabidopsis* lines.

*Arabidopsis* was transformed with the pCambia1300-*MeGLYI-13*-*GFP* vector. A total of six transgenic lines were generated under hygromycin selection, and the presence of *MeGLYI-13* was confirmed by PCR (Figure 4A). The six transgenic lines were named OE1, OE2, OE3, OE4, OE5 and OE6, respectively. To determine the *MeGLYI-13* gene expression in the transgenic lines, qRT-PCR was performed. The results revealed that the expression level of the *MeGLYI-13* gene varied in different transgenic lines. The transcriptional abundance of the *MeGLYI-13* gene was highest in the OE1 and OE5 lines, followed by OE2. The transcriptional abundance of *MeGLYI-13* was lowest in the OE4 transgenic line. There was no *MeGLYI-13* gene expression in the WT *Arabidopsis* (Figure 4B). Because the vector had a GFP tag to further verify the *MeGLYI-13* transgenic *Arabidopsis*, the seedlings of six transgenic *Arabidopsis* lines were exposed to a LUYOR-3415RG dual-wavelength fluorescent protein excitation light source. The results revealed that the leaves of the WT and OE4 with low *MeGLYI-13* expression were red, while the leaves and roots of the other five transgenic lines showed a strong green fluorescence. This indicated that the *MeGLYI-13* gene was successfully translated in lines OE1, OE2, OE3, OE5 and OE6 (Figure 4C).

### 2.6. MeGLYI-13-Overexpressing Arabidopsis Showed Better Tolerance to Zinc Shortage and Toxity

**Figure 5:**
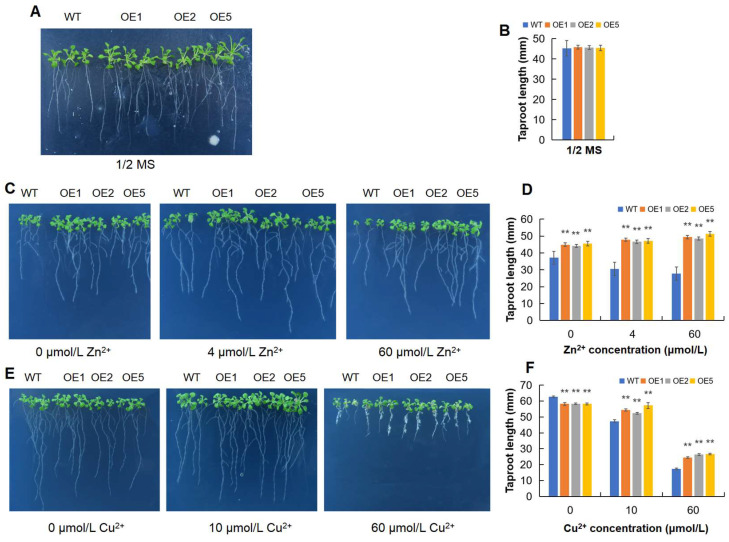
Zinc and copper tolerance analysis of *MeGLYI-13* transgenic *Arabidopsis*. (**A**) Phenotypes of *Arabidopsis* grown on normal 1/2 MS medium. (**B**) Statistics of the taproot length of *Arabidopsis* grown on normal 1/2 MS medium. (**C**) Phenotypes of *Arabidopsis* under different Zn^2+^ concentrations. (**D**) Statistics of the taproot length of *Arabidopsis* under different Zn^2+^ concentration treatments. (**E**) Phenotypes of *Arabidopsis* under different Cu^2+^ concentration stresses. (**F**) Statistics of the taproot length of *Arabidopsis* under different Cu^2+^ concentration treatments. The error lines represent ±SD, n = 3; ** indicates significant differences of *p* ≤ 0.01, respectively.

To test whether *MeGLYI-13* is involved in the tolerance to Zn^2+^ or Cu^2+^ toxicity, three independent lines—namely, OE1, OE2 and OE5—of *MeGLYI-13* transgenic *Arabidopsis* were selected for study. The wild type and *MeGLYI-13*-overexpressing in vitro-grown plants were separately treated with different concentrations of Zn^2+^ and Cu^2+^. This study revealed that, when grown on normal 1/2 MS medium (15 μmol/L Zn^2+^ and 0.05 μmol/L Cu^2+^), there was no significant difference in the phenotypes of the WT and *MeGLYI-13*-overexpressing lines (Figure 5A,B). However, following treatment with Zn^2+^, we found that, when there was no Zn^2+^ or only low levels of Zn^2+^ (4 μmol/L) in the medium, the growth of WT was inhibited compared to the *MeGLYI-13*-overexpressing lines. Indeed, the leaves were smaller, and the taproots and lateral roots were shorter than that of the transgenic lines. After exposure to high levels of zinc (60 μmol/L), the taproots of the WT were obviously shorter than those of the *MeGLYI-13*-overexpressing lines. The lateral roots of the WT were hard to cultivate, whereas those of the *MeGLYI-13*-overexpressing lines grew well. Meanwhile, the leaves of the WT were also smaller than those of the *MeGLYI-13*-overexpressing lines (Figure 5C,D). The above consequences indicated that the overexpression of *MeGLYI-13* enhanced the survivability towards both the deficiency and toxicity of zinc.

### 2.7. MeGLYI-13-Overexpressing Arabidopsis Showed Better Tolerance to Copper Toxity

When grown on zero Cu^2+^ medium, the taproot length of the *MeGLYI-13*-overexpressing lines was distinctly shorter than that of the WT. When grown on the medium containing 10 μmol/L Cu^2+^, by contrast, the roots of the *MeGLYI-13*-overexpressing lines became longer than those of the WT, and the leaves were visibly larger. At high concentrations (60 μmol/L), however, the growth of both the WT and *MeGLYI-13*-overexpressing lines were significantly inhibited. However, the *MeGLYI-13*-overexpressing lines still grew better than the WT, with larger leaves, longer taproots and more root hair (Figure 5E,F). Therefore, it is speculated that the *MeGLYI-13* gene is involved in Cu^2+^ tolerance.

## 3. Discussion

Zinc and copper are two essential metal elements for plant growth and development. The lack of these two elements in the environment can inhibit the growth of crops, resulting in, amongst other consequences, reduced yields and quality. However, the presence of excessive amounts of these two elements can also be toxic to plants [4]. In recent years, the extensive use of zinc-containing and copper-containing pesticides and fertilizers, as well as the development of zinc and copper mining, means that the pollution of zinc and copper in soil has become increasingly serious [3,37]. In order to solve the damage caused by soil metal pollution to plants, people have tried many methods. One approach is to use soil amendments to limit the migration of metal ions so as not to affect plant growth [20]. Another approach is to increase a plant’s tolerance to metal ion toxicity by spraying it with chemicals [5]. In addition, another method is genetic engineering to improve the metal ion tolerance of plants. The improved plants can efficiently absorb metal ions in the soil and reduce the excessive accumulation of metal ions. Therefore, it is important to cultivate plants resistant to zinc or copper toxicity for the phytoremediation of soil contaminated with copper and zinc. In this respect, a two-phase strategy is required: firstly, the generation of plants that can cope with heavy metals and produce sustainable yields and, secondly, finding a mechanism by which heavy metals are stored in the part of the plants not used for human and/or animal consumption [38,39]. Cassava is a kind of woody shrub, with its tuberous roots full of starch, which is a good resource for producing bioenergy. A previous study indicated that cassava could grow well in Pb-, Cd-, Zn- and Cu-polluted soils. The soil mobile metal and bioavailable metal content decreased significantly after cassava planting [36]. Thus, cassava can be used as an important plant resource for phytoremediation—on the one hand, to improve the polluted soil environment, and on the other hand, to produce bioenergy raw materials. Furthermore, the key genes for heavy metal resistance in cassava can also be used to improve heavy metal resistance in plants. 

To combat the stress of metal toxicity, plants have evolved various protective mechanisms. Since metal toxicity stress can induce cytotoxic MG production in plants, plants have evolved a glyoxalase system to detoxify intracellular MG. Some reports have shown that the physiological mechanism of chemical measures to improve heavy metal resistance in plants involves the mobilization of a glyoxalase system [22,23,29,32,34,35]. Glyoxalase I (GLYI) is in the first step of the glutathione-dependent glyoxalase pathway to scavenge excess MG into S-D-lactoylglutathione. GLYI family genes have been identified and characterized in many species, such as *Arabidopsis* [40], rice [19], sugarcane [41], Chinese cabbage [42], grapes [43] and date palms [44]. We have also identified 19 *GLYI* family gene members in cassava [33]. The function of some *GLYI* genes in abiotic stress has been continuously demonstrated by experiments. For example, a sugar beet M14 glyoxalase I gene could increase *E. coli* tolerance to MG and increase tobacco tolerance to MG, H_2_O_2_, salt and mannitol [45], whereas an *OsGLYI* gene from rice was proven to improve rice tolerance to NaCl, ZnCl_2_ and mannitol [46]. Similarly, a sugarcane *GLYI* gene (*SoGloI*) improved *E. coli* tolerance to NaCl, CuCl_2_, CdCl_2_ and ZnSO_4_ stress [47]. Mutants of nuclear-located *AtGLYI-2* displayed an increase in the level of MG, a decrease in GLYI activity and were more susceptible to salt stress [19], while nuclear-located *OsGLYI-8* could recue the sensitivity of the *atglyi-2* mutant to MG and salt stress [19]. In view of previous reports on the role of *GLYI* genes in plant regulation of abiotic stress resistance, especially resistance to heavy metals, and our previous finding that the cassava *MeGLYI-13* gene confers Fe^3+^ resistance [33], we speculate that *MeGLYI-13* may also confer other heavy metal stress resistance, which is worthy of further study. Here, in our study, we found that the expression of the *MeGLYI-13* gene was induced by Zn^2+^ stress, and the peak value appeared at 6 h in the leaves and at 24 h in the roots (Figure S2A). The expression of the *MeGLYI-13* gene was also induced by Cu^2+^ stress, and the peak value appeared at 12 h in the roots, stem and leaves (Figure S2B). The function of MeGLYI-13 in high-concentration Zn^2+^ and Cu^2+^ stress tolerance was further confirmed by gene overexpression in yeast cells and *Arabidopsis* seedlings (Figure 5). These results indicate that the *MeGLYI-13* gene has a potential role in broader heavy metal stress resistance. However, the specific mechanism by which *MeGLYI-13* enhances Zn^2+^ and Cu^2+^ resistance is still unclear. As evolutionarily ubiquitous metalloenzymes, GLYIs require bivalent metal ions to bind to their active sites to activate the enzyme [30]. The activity of different GLYI enzymes depends on different metal ions. For example, in rice, the OsGLYI-11.2 enzyme was activated by Ni^2+^ and Co^2+^ but not by Zn^2+^ [30], which is similar to two GLYIs (GLXI;1 and GLXI;2) in *Arabidopsis* [48]. In *Arabidopsis*, another GLYI (GLXI;3) could be activated by Mn^2+^, Co^2+^ and Zn^2+^ [48]. Whether Cu^2+^ plays a role in promoting GLYI activity is unknown. In our current study, we found that there were more than 10 binding sites for Zn^2+^ or Cu^2+^ in the MeGLYI-13 amino acid sequence, and there were six common binding sites for both (Figure 1). These consequences forecast that MeGLYI-13 has the binding potential of Zn^2+^ and Cu^2+^. Moreover, Zn^2+^ and Cu^2+^ both might have a role in MeGLYI-13 activity, but the degree to which they activate the MeGLYI-13 enzyme needs to be verified experimentally.

In this study, we tested the heavy metal tolerance function of the *MeGLYI-13* gene in model organisms of yeast and *Arabidopsis*, because these two organisms are commonly used in laboratory studies and are easy to manipulate. Of course, if other organisms have matured genetic transformation systems, they can also be used to produce transgenic lines that overexpress *MeGLYI-13* to repair soil contaminated by heavy metals. In addition, virus-induced gene silencing (VIGS) is a powerful tool to trigger transient sequence-specific gene silencing in plants, which is very helpful for studying gene function. VIGS techniques have also been reported in cassava but mostly for cassava leaves and less for the roots [49,50,51,52]. It would be helpful to silence the *MeGLYI-13* gene in SC8 cassava roots by VIGS to better understand the function of *MeGLYI-13* in the tolerance of plants to Zn^2+^, Cu^2+^ and other heavy metals. Whilst the ultimate proof of function needs to be provided in future experiments in which loss-of-function of the *MeGLYI-13* gene is assessed directly in cassava, the data obtained in *Arabidopsis* suggest that it is highly likely that this gene mediates the tolerance of plants to Zn^2+^ and Cu^2+^ toxicity.

In our study, the *MeGLYI-13* gene cloned from the SC8 cassava variety has one nucleotide difference and one amino acid difference compared to the sequence from the AM560-2 cassava variety (Figure S1). This result suggests that the *MeGLYI-13* gene may display genotypic differences. Therefore, it is necessary to conduct sequencing verification in the study of other genotypes of cassava. Interestingly, in rapeseed, the *BnGLYI-3* gene from a heat-tolerant rapeseed cultivar was found to improve the heat and cold tolerance of yeast cells, while the *BnGLYI-2* gene from a heat-sensitive rapeseed cultivar was not, although there are only two amino acid differences between the BnGLYI-2 and BnGLYI-3 sequences [53]. It can be seen that, sometimes, changes in a few amino acids can lead to large differences in gene function. Therefore, whether the difference in the sequence of cassava MeGLYI-13 will affect the function of the gene remains to be verified.

Protein subcellular localization is very important for the study of protein function. Onion epidermal cells are large, living, transparent monolayer cells, which are suitable for visual fluorescence fusion protein observation. The research presented in our study shows that MeGLYI-13 from SC8 cassava is located in the nucleus of onion epidermal cells (Figure 2). To our knowledge, MeGLYI-13 is the first glyoxalase I protein found to be localized in the nucleus in cassava. The nuclear location of MeGLYI-13 is suggestive of a regulatory role. A few reports also found nuclear localization of GLYIs, such as *AtGLYI2-4* in *Arabidopsis* and *OsGLYI-8* in rice [25]. Their nuclear localization signal (NLS) sequences were predicted and experimentally verified [25]. Meanwhile, MG has also been detected in the nucleus, which indicated that the MG detoxification mechanism presents in the nucleus. According to previous studies, we also used the same tool to predict a strong nuclear localization signal in the MeGLYI-13 amino acid sequence (Figure S3). These results confirmed the nuclear location of MeGLYI-13, suggesting that MeGLYI-13 may play a vital role in nuclear MG detoxification, protecting the nucleus from adverse stress.

It seems strange that MeGLYI-13 is localized in the nucleus and resistant to heavy metal ion toxicity. However, previous studies have confirmed that heavy metal ions can enter the nucleus after crossing the plasma membrane barrier, thus interfering with nucleic acids, chromatin and the internal components [54,55,56]. For example, a higher concentration of copper induced a change in the nucleus structure, higher chromatin density and lower DNA synthesis [56]. DNA damage was found in potatoes after cadmium treatment [55]. In order to reduce the damage of heavy metals to the nucleus, some genes in the nucleus actively respond; in addition to some common transcription factors, there are some other genes. For instance, cysteine synthase 1 (OsCS1) in rice was proven to be localized in the nucleus, and its overexpression transgenic plant obtained a higher tolerance to cadmium stress [57]. A rice heavy metal-associated isoprenylated plant protein gene (OsHIPP17) was localized in the nucleus, and overexpressing it in *Arabidopsis* enhanced the copper tolerance of transgenic plants [58]. Therefore, we speculate that MeGLYI-13 localization in the nucleus may be related to the clearance of excessive MG caused by heavy metals in the nucleus, and the binding sites for Zn^2+^ or Cu^2+^ may be used for binding Zn^2+^ or Cu^2+^ in order to activate MeGLYI-13 enzyme activity.

## 4. Materials and Methods

### 4.1. MeGLYI-13 Gene Analysis

The total RNA from 40-day tissue culture seedlings of SC8 cassava (*Manihot esculenta* Crantz) was isolated following the instructions of the Plant Total RNA Isolation Kit Plus (FOREGENE). The RTIII Super Mix (with dsDNase) kit (MonScript, Monad, Suzhou, China) was used to clear away possible residual DNA fragments in the total RNA solution, and after, the first strand of cDNA was synthesized. According to the sequence of Manes.12G054100 in the AM560-2 cassava genome data, Primer Premier 6.0 was used to design a pair of specific primers with a predicted length of 556 bp (Table S1). The 50 μL PCR system included PrimeSTAR HS (premix) 25 μL, template cDNA (1 μg/μL) 1μL, 10 μmol/L upstream and downstream primers 2 μL each of ddH_2_O 20 μL. The PCR procedure included 94 °C pre-degeneration for 5 min, 98 °C degeneration for 10 s, 55 °C annealing for 15 s, 72 °C extension for 1 min for 32 cycles and 72 °C extension for 10 min. PCR production was then detected using 1% agarose gel electrophoresis. The target fragment was purified by the use of a Gel Extraction Kit (Omega) and then sequenced by Sangon Biotech. The sequence alignment was performed using Jellyfish software version 4.1.1.0. The Zn^2+^ and Cu^2+^ binding sites were predicted by the online tool MIB2 (http://bioinfo.cmu.edu.tw/MIB2/, accessed on 21 November 2022). The nuclear localization signal on the MeGLYI-13 protein sequence was predicted by using the online tool cNLS Mapper (http://nls-mapper.iab.keio.ac.jp/cgi-bin/NLS_Mapper_form.cgi, accessed on 6 January 2023).

### 4.2. Subcellular Localization of MeGLYI-13

SnapGene software version 4.2.4.0 was used to design homologous recombination primers. *Spe*I restriction sites were introduced upstream and *Kpn*I restriction sites downstream of ORF of the *MeGLYI-13* gene. By using the Seamless Assembly Cloning Kit (Clone Smarter, USA), the fusion expression vector pCAMBIA1300-35S-*MeGLYI-13: GFP* was obtained. The vector was then transferred into DH-5α *E. Coli* for sequencing. Both the pCAMBIA1300-35S-*MeGLYI-13: GFP* vector and the control pCAMBIA1300-35S-*GFP* vector were transformed into living onion epidermal cells by an *Agrobacterium Tumefaciens* LBA4404-mediated protocol. After incubation at 28 °C on solid MS medium (pH5.8) for 16–24 h, the onion cells were studied using a Laser Scanning Confocal Microscope FV1000 (Olympus, Tokyo, Japan).

### 4.3. Expression Analysis of MeGLYI-13 in Response to Zinc and Copper Stress

To explore the response of the MeGLYI-13 gene in zinc or copper stress, 30-day-old cassava seedlings of the SC8 variety were treated with 60 µmol/L Zn(CH_3_COOH)_2_ or 100 µmol/L CuSO4 for 0, 2, 6, 12 and 24 h, respectively. Three plants and three biological replicates were set for each treatment. The leaves, roots and stems were harvested and used to extract RNA and perform a qRT-PCR analysis according to the method in our previous report [33].

### 4.4. Yeast Transformation and Stress Treatments

In order to construct a yeast expression vector, a restriction site (*Kpn*I) was introduced upstream of the *MeGLYI-13* ORF, and an additional restriction site (*EcoR*I) was introduced downstream of the gene. Afterward, a fragment of *MeGLYI-13* gene was inserted into the yeast expression vector pYES2 by the double restriction method to generate the pYES2-*MeGLYI-13* yeast expression vector. The sequences of the primers pYES2-*MeGLYI-13* forward (F) and pYES2-*MeGLYI-13* reverse (R) are given in Supplementary Table S1. The empty plasmid pYES2 and recombinant plasmid pYES2-*MeGLYI-13* were transformed into *E. coli* DH5α using the chemical transformation method, and the presence of the transgene was detected by PCR and subsequent sequencing. The pYES2 and pYES2-*MeGLYI-13* plasmids in *E. coli* DH5α with correct sequencing were extracted and transferred into INVSc1 yeast strains using the PEG/LiAc transfer method. In order to investigate the response of *MeGLYI-13* transgenic yeast to Zn^2+^ and Cu^2+^ ion stress, transgenic yeast and control yeast were cultured in SC-Ura liquid medium (2% galactose, 25 mg/L Amp) at 28 °C and 180 r/min for 36 h. The bacteria were then collected by centrifugation and then resuspended to OD600 = 0.2 by SC-Ura liquid medium containing different Zn^2+^ (0 mmol/L, 6 mmol/L, 12 mmol/L, 18 mmol/L, 24 mmol/L and 30 mmol/L) and Cu^2+^ (0 mmol/L, 0.6 mmol/L, 1.2 mmol/L, 1.8 mmol/L, 2.4 mmol/L and 3.0 mmol/L). Then, 2 mL of each bacterial solution was inserted into a new tube and incubated at 28 °C, 180 r/min for 30 h, and the OD600 value was measured. Three biological replicates were performed per treatment. At the same time, the survival difference between the recombinant yeast and the control yeast after 30 mmol/L Zn^2+^ and 3 mmol/L Cu^2+^ stress was assessed. Then, 5 mL of bacterial solution cultured for 36 h was centrifuged to collect the bacteria, respectively, and suspended in the treatment solution and cultured for 6 h at 28 °C, 180 r/min. After that, the treated bacterial suspension was attenuated into the six 10^−1^, 10^−2^, 10^−3^, 10^−4^, 10^−5^ and 10^−6^ gradient dilutions. Then, 5 μL of diluted bacterial suspension was plated on SC-Ura solid medium. The bacteria were cultured at 28 °C, and two days later, the survival difference of the yeast was observed and photographed.

### 4.5. Transformation of Arabidopsis with MeGLYI-13

The *Agrobacterium* LBA4404 containing the pCAMBIA1300*-MeGLYI-13:GFP* expression vector was shock cultured until the OD600 value of its suspension reached 0.9~1.0 at 28 °C and with 250 r/min shaking with YEP liquid medium containing 50 mg/L kanamycin and 50 mg/L rifampicin. The suspension was then centrifuged to collect the bacteria at 4000× *g* and 4 °C. After washing one time in 1/2 MS liquid medium (containing 0.03% Silwet-L77), the bacteria were resuspended to an OD600 value of 0.6~0.8 and then kept away from light for 3~12 h. Inflorescences containing unopened flowers of *Arabidopsis* were dipped into the above bacteria suspension for 3 min, then cultured in the dark for 16~24 h. After that, the *Arabidopsis* were cultured under 16 h light/8 h dark at 22 °C conditions for three days. The mature seeds were collected as the T0 generation. The seeds were disinfected with 75% ethanol (containing 0.01% Tween 20) for 5 min and washed by absolute ethanol twice. Next, the seeds were evenly spread on 1/2 MS solid medium (containing 80 mg/L hygromycin) and vernalized at 4 °C under darkness for two to four days and then cultured under 12 h light/12 h dark at 22 °C. Ten days later, the *Arabidopsis* seedlings that grew well on the resistance medium were transplanted into soil for pot culturing.

### 4.6. Detection of Transgenic Arabidopsis

To detect transgenic *Arabidopsis*, we performed DNA-level PCR. Then, 2 mm^2^ leaves of each *Arabidopsis* seedling were collected separately and treated with 20 μL lysate (M5 mix Kit, MF848, Mei5bio, Beijing, China) until the liquid turned green. The liquid was kept at 95 °C for 5 min and shortly centrifuged for 1 min at 10,000× *g*, and then, the supernatant was used as a template for the PCR. The sequences of primer pairs 1300F and 1300R used to detect the transgenic plants are given in Supplementary Table S1. The 20 μL PCR reaction liquid contained 10 μL 2 × M5 HiPer plus Taq HiFi, 0.5 μL 1300F, 0.5 μL 1300R, 2 μL template DNA and 7 μL ddH_2_O. The PCR procedure included 95 °C for 3 min, 94 °C for 25 s, 53 °C for 25 s, 72 °C for 45 s for 34 cycles and a 72 °C extension for 5 min. The PCR product was detected by 1% agarose electrophoresis.

### 4.7. Expression Level Detection of MeGLYI-13 in Transgenic Arabidopsis

To detect the expression level of *MeGLYI-13* in PCR-positive lines by qRT-PCR, the total RNA from the PCR-positive *Arabidopsis* lines was extracted, purified and reverse-transcribed by successively using the RNA Isolation Kit (Foregene, Chengdu, China) and RTIII Super Mix with dsDNase kit (Monad, Suzhou, China). To perform the reaction for qRT-PCR, the reaction system was prepared according to the manual of SYBR^®^ Premix Ex Taq^TM^ II (Takara, Otsu, Japan). For detecting the *MeGLYI-13* gene, the primer pairs of QMeGLYI-13F and QMeGLYI-13R were used. For reference, the *Actin* gene primer pairs ActinF and ActinR were used (see Supplementary Table S1). QRT-PCR was performed and recorded using a 7900 HT Fast Real-Time PCR System (ABI, Foster City, CA, USA). The relative expression levels of *MeGLYI-13* in different *Arabidopsis* lines were calculated using the 2^−∆∆CT^ method. The wild type *Arabidopsis* (WT) was used as the control. Three biological replicates were performed in this experiment.

### 4.8. Fluorescence Detection of Transgenic Arabidopsis

The T1 generation seeds were germinated on 1/2 MS medium after vernalization, as described in Section 2.4. Then, a dual-wavelength fluorescent protein excitation light source (Luyor-3415RG, Luyor Corporation, Shanghai, China) was used to irradiate the germinated seedlings. Positive transgenic *Arabidopsis* seedlings were selected, as they showed green fluorescence under light, while the wild type and transgenic lines with too-low gene expression showed a red color [59].

### 4.9. Znic and Copper Stress Treatment of Transgenic Arabidopsis

After identification by PCR, qRT-PCR and fluorescence, some transgenic lines were selected for further experiments. The WT seeds and T4 generation seeds of three selected transgenic *Arabidopsis* lines were sterilized and seeded on 1/2 MS medium at 4 °C for 2 days of vernalization and then germinated at 22 °C for 12 h light/12 h dark for 8 days of germination. Then, the *Arabidopsis* seedlings were transferred to 1/2 MS medium containing diverse concentrations of Zn(CH_3_COOH)_2_ (0, 4 and 60 μmol/L) and CuCl_2_ (0, 10 and 60 μmol/L) for stress treatment under 22 °C for 12 h light/12 h dark cultural conditions for 12 days. The normal 1/2 MS medium (Zn^2+^ 15 μmol/L and Cu^2+^ 0.05 μmol/L) was set as the control. After treatment, any phenotypic changes were observed and photographed. The length of the taproot was measured. For each treatment, three plates were set up, and three plants were used per line in each plate.

### 4.10. Statistical Analysis

All data analyses and graphic output were performed using GraphPad Prism 8.0 software. The one-way analysis of variance was performed to analyze the data, and Dunnett’s multiple comparisons test was used to assess the significant differences.

## 5. Conclusions

In summary, a *MeGLYI-13* gene was cloned from SC8 cassava with a cDNA length of 495 bp and protein length of 164 amino acids. The *MeGLYI-13* from SC8 cassava germplasm was different to that from AM560 cassava germplasm with one nucleobase difference in the cDNA sequence and one amino acid in the protein sequence. Subcellular localization of the MeGLYI-13 protein was demonstrated in the nucleus. There, six binding sites were predicted to bind to both Zn^2+^ and Cu^2+^ in the MeGLYI-13 protein sequence. The overexpression of *MeGLYI-13* enhanced the Zn^2+^ and Cu^2+^ toxicity in both yeast cells and *Arabidopsis* seedlings. The results of this study highlight the function of one *MeGLYI-13* gene from cassava in resistance to multiple heavy metals stress, possibly by stabilizing the MG balance in the nucleus. It can thus likely be used as a target for genetic modification to improve crop resistance to heavy metals. Therefore, we will continue to explore the mechanisms by which *MeGLYI-13* regulates Zn and Cu toxicity tolerance in the future.

## Data Availability

The datasets generated during and/or analyzed during the current study are available from the corresponding author on reasonable request.

## Supplementary Materials

The following supporting information can be downloaded at https://www.mdpi.com/article/10.3390/plants12193375/s1: Figure S1: Sequence alignments of *MeGLYI-13* CDS (A) and amino acids (B) between SC8 and AM560-2 cassava genome data. Figure S2: Expression profile of *MeGLYI-13* in cassava seedlings after 60 μmol/L Zn^2+^ and 100 μmol/L Cu^2+^ treatments. Figure S3: Nuclear localization signal (NLS) prediction of MeGLYI-13 in cassava SC8. The red characters indicate the NLS sequence. Table S1: Sequences of the primers.
