# Supplementary material for: MeGLYI-13, a Glyoxalase I Gene in Cassava, Enhances the Tolerance of Yeast and Arabidopsis to Zinc and Copper Stresses
Source: Plants (Basel). 2023 Sep 25;12(19):3375. doi: 10.3390/plants12193375 (PMC10574700; doi:10.3390/plants12193375)
Supplement: Supplementary file 1 [file plants-12-03375-s001.zip › Figure S.pdf]

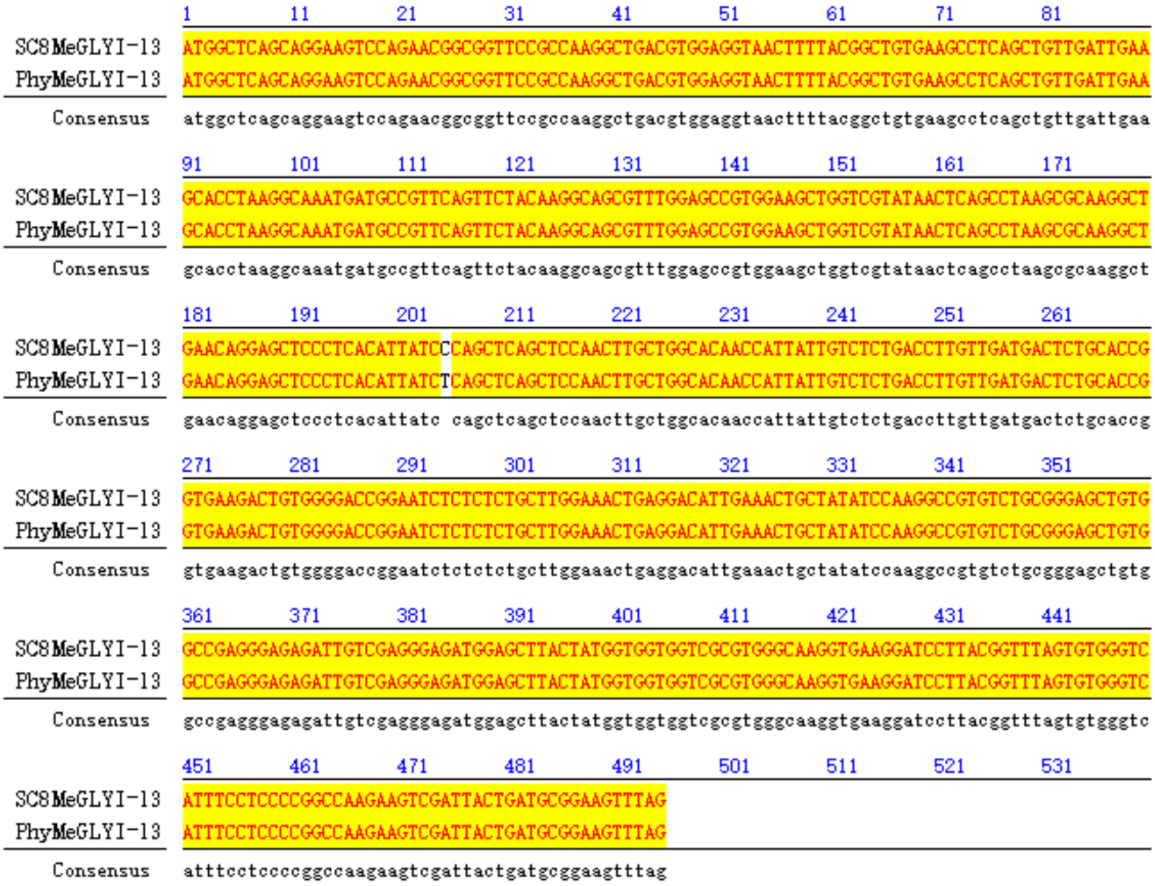

A

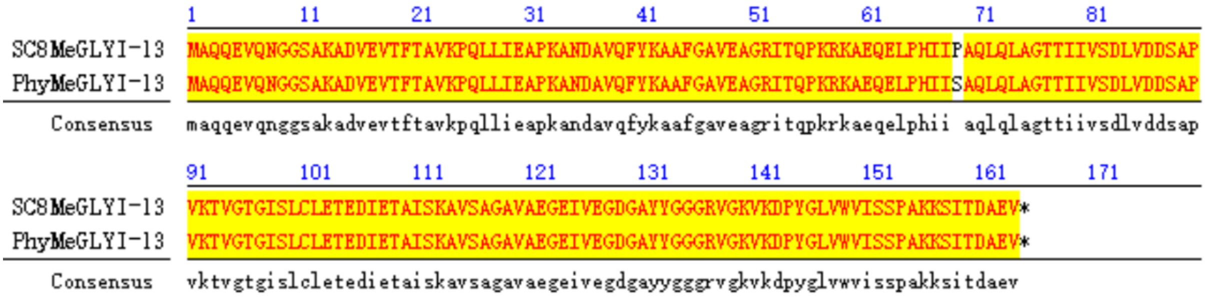

B

Figure S1 Sequence alignments of *MeGLYI-13* CDS (A) and amino acid (B)between SC8 and AM560-2 cassava genome data

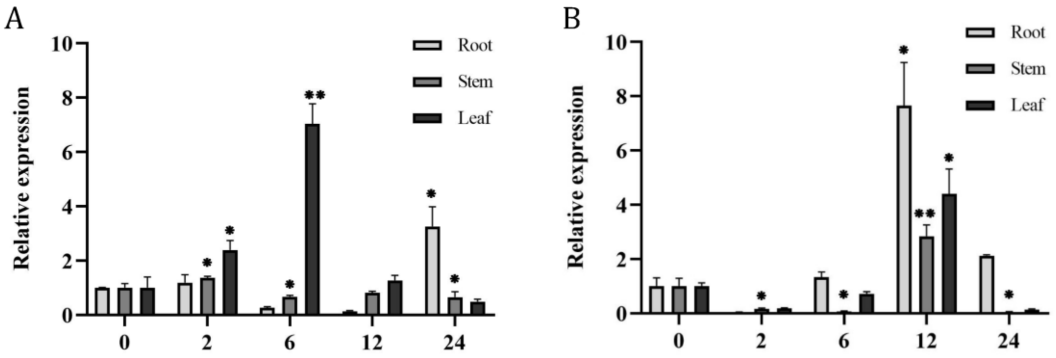

Figure S2 Expression profile of *MeGLYI-13* in cassava seedlings after 60 μmol/L Zn^{2+} (A) and 100 μmol/L Cu^{2+} (B) treatments. Velues are means and standard deviations (n = 3). * indicates the significant difference $p \leq 0.05$, ** indicates the significant difference $p \leq 0.0$.

cNLS Mapper Result

Predicted NLSs in query sequence	
GLYMAQQEVQNGGSAKADVEVTFTAVKPQLLIEAPKANDAVQFYKAAFGA	50
VEAGRITQPKRKAEQELPHIIPAQLQLAGTTIIVSDLVDDSAPVKTVGTG	100
ISLCLETEDIETAISKAVSAGAVAEGEIVEGDGAYYGGGRVGKVKDPYGL	150
VWVISSPAKKSITDAEV	167

Predicted monopartite NLS		
Pos.	Sequence	Score
57	TQPKRKAEQE	5

Figure S3 Nuclear localization signal (NLS) prediction of MeGLYI-13 in cassava SC8. The red characters indicate the NLS sequence.
